# A 3D color model reports urine color similarly to a printed color chart with similar accuracy to determine a low vs. high urine concentration

**DOI:** 10.1186/s44410-025-00009-3

**Published:** 2025-09-12

**Authors:** Raul Freire, Kinta D. Schott, Brooke Butterick, Zack Stow, Parker Kooima, Sai Tejaswari Gopalakrishnan, Emily Dow, Jason C. Siegler, Jefferey L. Burgess, Floris C. Wardenaar

**Affiliations:** 1 Athleat Field Lab, College of Health Solutions, Arizona State University, Phoenix, AZ 85054 USA; 2 Integrative Human Performance Lab, College of Health Solutions, Arizona State University, Phoenix, AZ 85054 USA; 3 Mel and Enid Zuckerman College of Public Health, University of Arizona, Tucson, AZ 85724 USA

**Keywords:** Hydration status, Athletes, Wildland firefighters, Validity study, Urine specific gravity, Urine osmolality, 24-h urine

## Abstract

**Background:**

Urine color (Uc) assessment is impacted by container, volume, and light conditions, potentially influencing Uc scoring. This study aimed to evaluate a 3D Uc model vs. Printed Uc chart’s accuracy for identifying a low vs. high urine concentration in the morning and afternoon.

**Results:**

Participants (*n* = 73, 12% female, age: ~ 27 years) collected all urine voids for two consecutive days (32 h total), resulting in two partially concurrent 24-h urine collections that were compared with a morning and afternoon assessment. Median and [interquartile range] Uc differed between the Printed chart and 3D Uc model in the morning (2.0 [1.5–3.0] vs. 2.0 [2.0–4.0], *p* < 0.001), but not in the afternoon (2.0 [1.0 – 3.0] vs. 2.0 [1.0 – 3.0], *p* = 0.07). The models had a moderate correlation in the morning (*r* = 0.66, *p* < 0.001) and a strong correlation in the afternoon (*r* = 0.83, *p* < 0.001). Bland–Altman plots revealed a slight bias (-0.60 and -0.27 for morning and afternoon, respectively), with significant reporting bias only for the morning, *p* = 0.04. The Area Under the Curve (AUC), to correctly classify urine concentration, was poor for morning Printed (0.62) and 3D (0.59) vs. fair for afternoon Printed (0.78) and 3D (0.74) for USG 24 h. Interestingly, the AUC for spot urine sample color was high (i.e., morning: 0.84 and 0.79), and afternoon: 0.91 and 0.92) for the Printed chart and 3D Uc model, respectively.

**Conclusions:**

The printed chart and 3D Uc model presented similar scores but with slightly higher accuracy in the afternoon.

**Supplementary Information:**

The online version contains supplementary material available at 10.1186/s44410-025-00009-3.

## Introduction

Several methods have been suggested to measure hydration status in the human body, including the direct assessment of fluid loss through dehydration (e.g., bodyweight change over time) [1, 2] and measuring plasma or urine concentration [2]. While lacking a gold standard method to measure total body water [3], one of the ways to look at hydration is to assess 24-h urine concentration, allowing for monitoring changes in fluid intake [4], to reflect hydration status. A practical limitation is that the analysis of 24-h urine osmolality, which is to be considered the standard [5], depends on high-cost equipment, and although analytic costs can be reduced by applying urine specific gravity, both methods still require trained professionals to perform the analyses [6]. A low 24-h fluid intake has inversely been related to a high urine concentration [7]. To measure urine concentration, multiple options are available, including osmolality, urine specific gravity (USG), and urine color (Uc). Whereas osmolality provides the most accurate measurement, the sensitive equipment is normally only found in laboratories [2], USG is likely more applicable for practitioners [7], and outside clinical settings Uc can be reasonably used to estimate urine concentration [8, 9]. Both USG and Uc show a good correlation with urine osmolality [8]. As 24-h collections to determine hydration have shown to be cumbersome, the collection of spot urine samples has been suggested [10]. Specifically, the collection of a spot urine sample in the afternoon has been demonstrated as a good proxy of a 24-h urine sample [10, 11], but more research is needed to confirm if Uc assessment reflects this finding in applied settings. Moreover, Uc charts allow people to assess their hydration status more frequently than lab-based assessments at low to no cost, which can help to track the hydration status more often and avoid underhydration [12]. In an applied setting, an individual would determine the color of their urine sample against a Uc chart, preferably applying a validated predetermined urine color cut-off value to predict if the concentration of the urine sample is below or above a certain urine concentration [12].

However, the assessment of Uc is impacted by factors such as the container used (volume and material [i.e., cap color and plastic or glass]) and light conditions (light type and intensity) [13]. For example, our lab previously tested the impact of type and light intensity on determining the urine concentration scores, finding that brighter LED light conditions (1848 lm per square meter [lm/m^2^]) increased the ability to discriminate low from high urine concentration samples (accuracy of 79% using the sample-over-chart method) [13]. The sample’s container volume also influences how the Uc is perceived, as using a 90 mL container results in a one-shade-darker Uc compared to a 30 mL container [13]. Further, it could be that the perception of printed colors could be different than the perception of the colors of a liquid sample; a liquid 3D urine color model would specifically target that last part. The three-dimensional model to be tested in this study consists of seven 30 mL tubes with a colored liquid inside, matching the seven colors of the printed color chart version [9]. It is presumable that this model can help to diminish the limitations that come with printed Uc scoring, as it might eliminate the difference in how the color of the actual urine sample and the reference is perceived as container material and size, and the way light impacts the perceived color of the solution can be standardized. It has been reported ealier that athletes rated Uc samples lighter than the actual color shade on a printed Uc chart (ICC = 0.30) [14], resulting in a higher number of false negative cases (when a darker sample is rated as lighter), as such the athlete may not take the appropriate action to drink more fluids. While small numerical changes (e.g., from Urine color 3 to 2) may seem minor, they can represent meaningful shifts from a urine specific gravity above vs. below the selected cut-off for this study of ≤ 1.012, especially in the context of daily monitoring or when guiding behavior change in athletes. These cutoffs are often used in applied sports settings to trigger action (e.g., increasing fluid intake), making even seemingly modest changes practically relevant.

Therefore, this study aimed a) to determine the accuracy of the Printed chart and 3D Uc model for identifying a low vs. high urine concentration of 24-h urine samples, as well as for spot morning and afternoon samples, and b) to compare the Uc score reported based on a newly developed 3D Uc model and a previously validated printed Uc chart.

## Methods

### Design

**Fig. 1:**
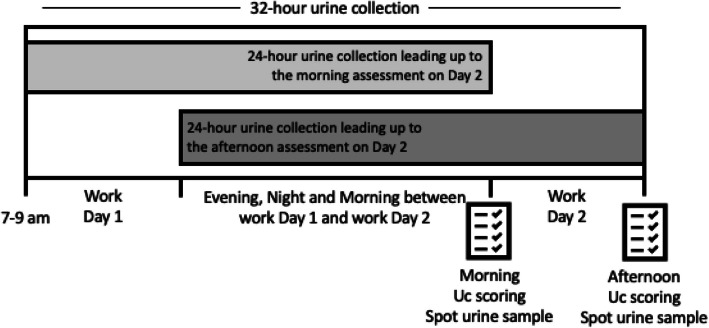
Timeline for urine collection illustrating the overlap of the 24-h urine samples used against morning and afternoon assessment

For this study, performed from March to October 2024, a new 3D Uc model was compared to a previously validated printed 7-color Uc chart [9] and against urine concentration criteria to predict a low vs. a higher urine concentration as part of a larger data collection assessing the accuracy of a wide range of hydration self-assessments. Participants collected every urine void in individual containers for a total of 32 h, starting between 7–9 am on the morning of Day 1 and finishing at 3–5 pm on Day 2 (Fig. 1). Urine volume and concentration for the first 24 h leading up to the morning assessment on Day 2, as well as 24-h urine collection leading up to the afternoon assessment on Day 2 were calculated and measured. The two 24-h urine samples overlapped, as the last 16 h of the first 24-h urine collection were the same as the first 16 h of the second 24-h urine collection. This allowed for comparing the morning and the afternoon self-assessment with the real 24-h urine concentration leading up to each measurement. This approach ensured that the concentration of the two 24-h samples could be measured. Then, the morning assessment was compared with the first 24-h urine collection concentration, and the afternoon concentration was compared with the second partially concurrent 24-h urine collection. The spot morning and afternoon urine samples were analyzed for color using the two different self-assessment methods (Printed and 3D), and the Uc outcome was compared to the urine concentration from the 24-h urine samples and the spot urine samples.

### Participants

A total of 61 wildland firefighters and 22 recreational athletes volunteered to participate in the study. The aimed sample size (*n* = 82) was based on a known estimated underhydration incidence in a general athletic population of 35% [15] and an expected maximal underhydration level of 50% within this active population, with an alpha of 0.05 and power of 80%.

The Institutional Review Board of Arizona State University (STUDY00018531) and the Department of Homeland Security (DHS) Compliance Assurance Program Office (DHS Regulatory Compliance Assessment: HSR-23–088) approved the study. Participation was voluntary, and participants read and signed informed consent before the start of the study. Due to federal regulations, no incentives were provided to wildland firefighters. The recreational athletes received a $50 digital gift card.

### Procedures

**Fig. 2:**
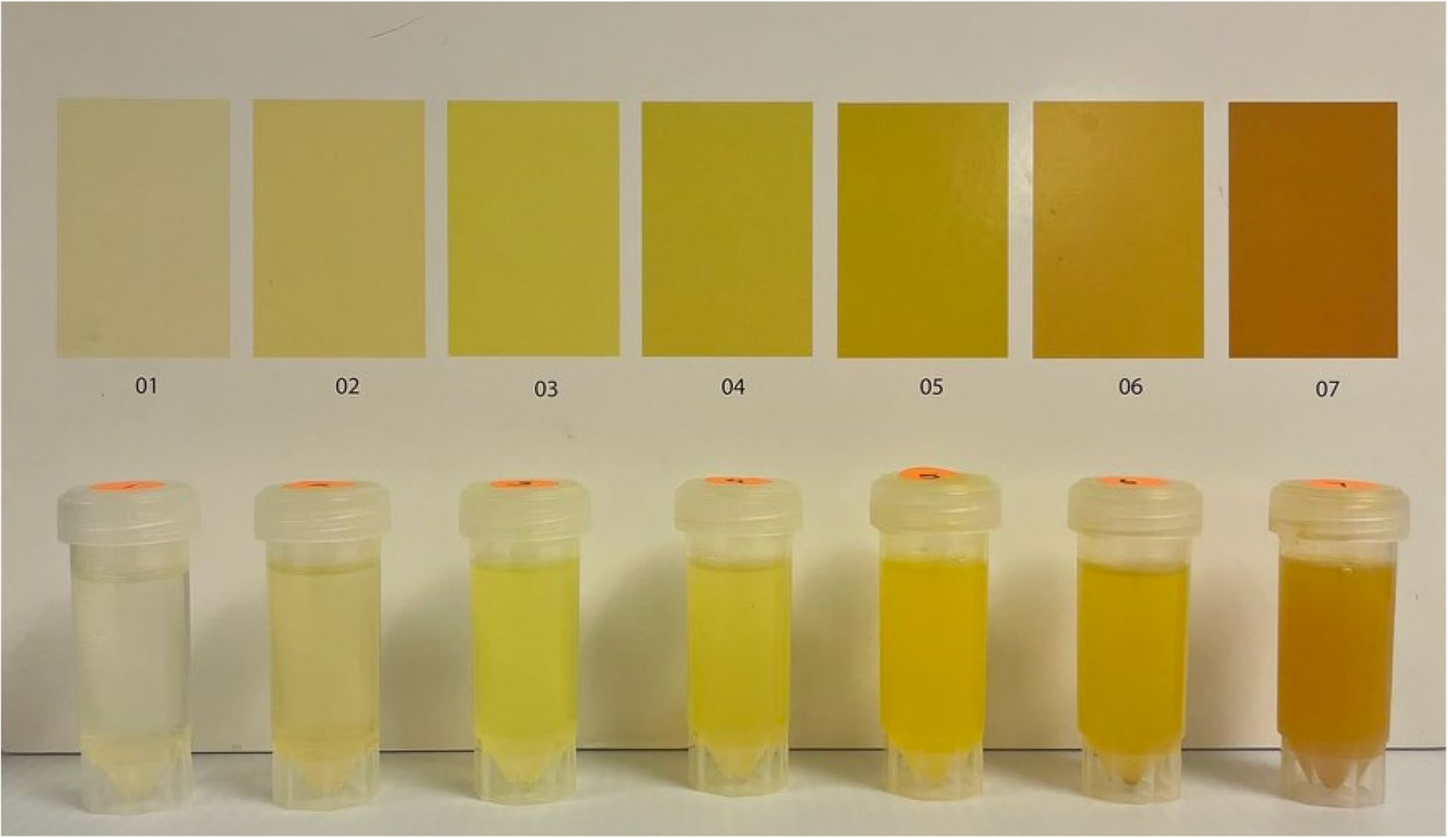
Printed 7-color chart and color-matched 3D color model used in the study

Throughout the study, urine collection started between 0700 and 0900 h and finished the next day between 1500 and 1700 h. As Uc tends to deteriorate quickly [16], each urine sample used for color scoring was collected within an hour of the morning or afternoon self-assessment. These urine samples were collected in a black container to blind participants from seeing the color of their urine before the actual Uc assessment. Although data were collected at multiple locations, the morning and afternoon Uc assessments were performed under the same environmental conditions (i.e., room light exposure), as described in the *Measurements* section below. Light intensity at each testing location was measured using a foot-candle lux meter (Extech 407,026, Extech Instruments, Waltham, MA, USA) at the Tungsten/Daylight setting to ensure comparability between morning and afternoon conditions. All Uc assessments were performed with a moderate lux ranging from 350–600. The 3D Uc model and Printed Uc chart (Fig. 2) were shown to the participants in an alternate order to avoid any order bias. Uc scores based on both tools were compared to individual 24-h samples while being categorized as having a low vs. high urine concentration to determine euhydration as proposed in the literature [3, 9, 17]. The urine concentration was determined by 1) Urine specific gravity [USG] for each 24-h sample, and 2) the USG of the spot morning and spot afternoon samples.

### Printed color chart

The 7-color chart was developed based on USG, ranging from very low concentration (< 1.017, score 1) to very high concentration (> 1.031, score 7). Seven colors represented the range from low to high urine concentration expressed as hexadecimal and RGB codes (Color 1: 7499 C [F1E6B2]; Color 2: 600 C [F1EB9C]; Color 3: 602 C [F0E87B]; Color 4: 603 C [EDE04B]: Color 5: 605 C [E1CD00]; Color 6: 7758 C [D4C304], and Color 7: 124 C [EAAA00)). The colors were printed on glossy paper (43.2 × 27.9 cm) in rectangles measuring 5.1 × 7.6 cm. The full description of the printed color chart used can be found in a previous publication [9].

### 3D Uc model development

A 3D color model was developed to match the colors of the previously described printed color chart. Four different pigment-based watercolors- yellow light, yellow medium, gambage, and ochre (Dr. Ph. Martins, Oceanside, CA, USA) were mixed (when needed) and diluted in water to build up a color similar to the printed color chart. The Dr. Ph. Martins watercolor brand was chosen as it is available worldwide. Table 1 shows the recipe containing the colors used (and their quantity, measured in drops, each drop equaling ~ 0.04 mL) and the amount of water in which the ink was diluted. Then, a sample of 30 mL was transferred to a smaller container and sealed with a transparent cap (30 mL Centrifuge Tubes and Caps, Caplugs Evergreen, Buffalo, NY, USA). Colors were matched to the printed color chart in a room with fluorescent light, with an intensity of ~ 400 lx. Match results were confirmed by four experienced researchers, who provided feedback on the color matching while assessing the color samples against a white backdrop. They could also pick the color samples up and evaluate them in the air as well as against a white backdrop until a consensus was reached.

**Table 1:** Dilution factor for the seven colors developed in the present study using different pigment watercolor paints diluted in water

	Yellow light	Yellow Medium	Gambage	Ochre	Amount of water
	(drops of ink)				**(**mL)
Color 1	---	---	---	1	250
Color 2	---	---	---	6	500
Color 3	---	1	---	1	100
Color 4	1	---	---	8	100
Color 5	---	1	3	9	800
Color 6	---	---	---	4	100
Color 7	---	---	---	10	100

### Measurements

#### Printed color chart

The participants used the Sample Over Chart method [13] to determine the color that best represented the urine sample. During this method, the participant directly compared their urine sample with each color on the 7-color Uc chart while sliding the sample over a white backdrop. To control for lighting, a flashlight with six LEDs was provided (Ozark Trail, Ozark, AR, USA), giving 1848 lm/m^2^ when filtered by a single layer of white masking tape, as described earlier [13]. The flashlight was held directly underneath a 30 mL centrifuge tube while holding the tube and flashlight in the dominant hand. They then decided on the color of the sample while writing down the color’s number on a recording sheet.

#### 3D color model

The seven numbered (1–7) 30 mL centrifuge tubes were lined up in a white rack from brighter (left side) to darker (right side) and put on the table. The participants were instructed to choose the tube that best represented the urine sample color, which was also placed on the table. Participants were allowed to take the tubes out and put them side by side to compare the samples from different angles and make a decision. After deciding on the color, they wrote the color’s number on a recording sheet.

#### Urinalysis

Urine specific gravity (USG) was measured for the 24-h urine samples and both spot urine samples at the end of each Uc assessment day using a refractometer (PAL Athlete, Atago, Tokyo, Japan) in a thermoneutral environment (20–25 °C), within one hour after being collected. The spot urine samples used for the urine color assessment for both the printed Uc chart and the 3D model were collected in the morning (end of first 24 h) and afternoon (end of second 24 h) during (Fig. 1). The measurements were taken in duplicates. If measurements differed by more than 0.0005 between two measurements, a third measurement was performed, and the median was selected.

The cut-off for USG was set at ≤ 1.012. Outcomes below the lowest cut-off values indicated a low urine concentration, suggesting being well-hydrated by consuming a sufficient amount of fluid [19]. In contrast, outcomes above the highest cut-off values indicate a higher urine concentration, potentially due to a relatively low daily fluid intake. Additionally, not included in the results, Supplementary Table 1 reports the accuracy of the comparison of Uc scoring against higher cut-off values (i.e., ≤ 1.020 USG), with samples scoring above this cut-off value suggesting hypohydration [3].

### Statistical analysis

The statistical analysis was performed based on 73 participants (out of *n* = 85, 86%) after removing participants with missing data. In total, 12 participants did not provide the spot urine sample (morning or afternoon, when the Uc assessment was performed), which resulted in missing data, and consequently, exclusion from the study. Data normality was evaluated using the Kolmogorov–Smirnov test, histograms to determine kurtosis and/or skewness and Q-Q plots.

To address aim (a), receiver operating characteristics (ROC) curves were calculated for both Uc assessment methods while applying this to the 24-h USG, as well as for the spot morning and spot afternoon samples. For each outcome, the area under the curve (AUC), sensitivity, specificity, optimal urine concentration threshold score, and correct urine concentration scores (%), accuracy in classifying a low vs. high urine concentration while applying a low cut-off value (≤ 1.012 USG), was determined. The AUC was interpreted as ‘excellent’ (AUC ≥ 0.90), ‘good’ (AUC 0.80–0.89), ‘fair’ (AUC 0.70–0.79), or ‘poor’ (AUC 0.60–0.69). Sensitivity and specificity scores are preferred to be above 0.80 [20]. Sensitivity is the number of TP (True Positive) scores suggesting underhydration divided by the sum of TP (True Positive) and FN (False Negative) scores. Specificity is the number of TNs (True Negative) divided by the sum of FPs (False Positive) and TN. To classify urine samples with a low vs. high concentration, the optimal Uc threshold score to predict underhydration was determined from the AUC using the max approach of the sensitivity and specificity matching the selected urine concentration cut-off values. For instance, if the subject used the 3D model in the morning while applying the USG cut-off value ≤ 1.012 to categorize the 24 h urine as low vs. high, a Uc 1–4 would suggest a USG value ≤ 1.012, and if this was the case the analysis would be classified as True Positive. In case the subject rated the Uc as 5, while having a USG value of 1.013 or higher, the outcome would be classified as a False Positive.

To address aim (b), general characteristics for Uc scores were reported as the median and interquartile range (IQR). A Wilcoxon Signed Rank test was used to determine the mean difference between methods, with a statistical significance level set at *p* < 0.05 for all analyses performed. Spearman correlation coefficients were calculated to evaluate the association between the Printed Uc chart and the 3D Uc model, and 95% CI was calculated using Fisher’s Z transformation. Cohen’s *d* was used to evaluate the strength of Spearman’s correlation, indicating effect size: *small* (0.10–0.29), *medium* (0.30–0.50), and *large* (> 0.50) [21]. Finally, a Bland–Altman plot was constructed (with on the y-axis the difference between reported Uc outcomes vs. the x-axis reporting the means of both outcomes) to determine the bias and level of agreement (1.96 SD) between both tools. Furthermore, Spearman correlations were calculated for the x-axis and the y-axis of the Bland–Altman plot (e.g., the mean score of the two Uc methods vs. the difference between the two Uc methods) to assess data heterogeneity, in which a significant correlation suggests reporting bias.

## Results

A total of 85 participants participated in the study, but the results are based on a sample size of 73 participants (of which 12% were females), since some participants could not provide all the required urine samples at the appropriate time, resulting in missing data. Most participants were male (88%), and the average age was 27 (19–35) years old. The data for body mass, height, osmolality, USG 24 h, and USG spot samples were normally distributed, whereas Printed and 3D Uc model scores in the morning and afternoon were not. Considering the USG threshold (≤ 1.012), there was a difference between the 24-h urine collections, resulting in 58.9% and 56.2% of the samples being below the threshold for the first and second 24-h samples, respectively. Considering the spot samples, 24.7% and 50.7% were below the threshold for morning and afternoon samples, respectively. Table 2 shows the descriptive data for demographics, Uc and urine specific gravity. No statistical differences were found between the 24-h urine collections for USG (1st 24-h: 1.011 [1.004–1.018] and 2nd 24-h: 1.012 [1.004–1.020]), with *p* = 0.34. The USG spot samples (morning: 1.018 [1.008—1.028] and afternoon: 1.011 [1.000–1.026]) were borderline significant with *p* = 0.05). There was a small non-significant correlation between the 24-h USG value and the spot morning sample USG used for the 1 st 24-h morning assessment on Day 1 (*r* = 0.22, *p* = 0.07), and a good correlation for the 2nd portion of 24-h USG value and the spot afternoon USG sample leading up to the afternoon assessment on Day 2 (*r* = 0.66, *p < *0.001).

**Table 2:** Demographics, Uc scores, and urine concentration from *n* = 73 participants

	Median (IQR)	Min – Max	Significant difference
Age (years)	27 (19–35)	19–45	---
Body Mass (kg)	80.0 (62.5–97.5)	49.8–125	---
Height (cm)	176 (164–187)	159–190	---
USG 1^st^ 24 h	1.011 (1.004–1.018)	1.003–1.034	t = 0.95, *p* = 0.34
USG 2^nd^ 24 h	1.012 (1.004–1.020)	1.004–1.032	
USG Spot Morning	1.018 (1.008–1.028)	1.002–1.035	t = 2.00, *p* = 0.05
USG Spot Afternoon	1.011 (1.000–1.026)	1.001–1.032	
Printed color chart morning	2 (1.5–3.0)	1–6	Z = −3.99, *p* < 0.001
3D Uc model morning	2 (2.0–4.0)	1–6	
Printed color chart afternoon	2 (1.0–3.0)	1–6	Z = −2.70, *p* = 0.07
3D Uc model afternoon	2 (1.0–3.0)	1–6	

Data are presented as median ± interquartile range (IRQ) and Minimum and Maximum values

*USG* urine specific gravity, --:N/A. Significant differences were assessed using Wilcoxon Signed Rank Tests for Uc and paired t-tests for USG 24 h and USG spot, with *p* ≤ 0.05

### Comparison of Uc scores between printed and 3D Uc model

The self-reported median Uc for the Printed chart and 3D Uc model was significantly different in the morning (2.0 [1.5–3.0] vs. 2.0 [2.0–4.0], respectively, with Z = −3.99, *p* < 0.001), but not in the afternoon (2.0 [1.0–3.0] vs. 2.0 [1.0–3.0], respectively, with Z = −2.70, *p* = 0.07).

In the morning, Printed and 3D tools presented a good correlation (*r* = 0.66, *p* < 0.001, CI: 0.50—0.77). In addition, the correlation for afternoon scores showed a very strong correlation (*r* = 0.83, *p* < 0.001, CI: 0.74—0.89).

**Fig. 3:**
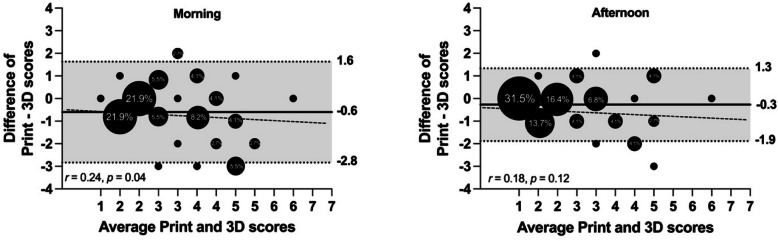
Bland & Altman plots comparing printed and 3D Uc scores in the morning and afternoon. The solid line represents the bias between printed and 3D Uc scores. The grey area represents the upper and lower limits of agreement (Mean difference ± 2 SD). The smallest dots represent 1.4% of the total sample

Finally, both Bland & Altman plots showed a slight mean bias (−0.54 and −0.24 for morning and afternoon, respectively) (Fig. 3). Plots showed a presence of data heterogeneity in the morning, indicating that in a darker color, the 3D Uc model overestimates the Printed version (*r* = 0.24, *p* = 0.04). On the other hand, the afternoon analysis did not present this characteristic (*r* = 0.18, *p* = 0.18).

### Receiving operator characteristic analyses

Overall, the Printed and 3D tools provided similar accuracy in determining a low vs. high urine concentration based on the 24-h urine samples in the morning (Table 3). For USG, Morning Printed AUC was 0.62, with an accuracy of 61.6%, vs. Morning 3D AUC of 0.59 and an accuracy of 60.3%. Interestingly, accuracy for the assessment of spot USG samples was good, with accuracy parameters being slightly better for Printed (AUC: 0.84, accuracy: 83.6%) vs. the 3D Uc model (AUC: 0.79, accuracy: 67.1%).

**Table 3:** Receiver operating characteristic evaluation of the Printed chart and 3D Uc model and percentage accuracy of correct classification of low vs. high urine concentration using lower cut-offs

Category	Diagnostic standard	Threshold	AUC	Sensitivity %	Specificity %	Accuracy %*	TP % and n	TN % and n	FP % and n	FN % and n	Uc Cut-off
Morning Print	USG 1^st^ 24 h	≤ 1.012	0.62	56.7	34.9	61.6	23.3 (17)	38.4 (28)	20.5 (15)	17.8 (13)	≤ 3
	USG spot morning	≤ 1.012	0.84	89.1	55.8	83.6	67.1 (49)	16.4 (12)	8.2 (6)	8.2 (6)	≤ 2
Morning 3D	USG 1^st^ 24 h	≤ 1.012	0.59	43.3	27.9	60.3	17.8 (13)	42.5 (31)	16.4 (12)	23.3 (17)	≤ 4
	USG spot morning	≤ 1.012	0.79	58.2	52.6	67.1	43.8 (32)	23.3 (17)	1.4 (1)	31.5 (23)	≤ 3
Afternoon Print	USG 2^nd^ 24 h	≤ 1.012	0.78	84.4	31.7	75.3	37.0 (27)	38.4 (28)	17.8 (13)	6.8 (5)	≤ 2
	USG spot afternoon	≤ 1.012	0.91	88.9	67.3	83.6	43.8 (32)	39.7 (29)	11.0 (8)	5.5 (4)	≤ 2
Afternoon 3D	USG 2^nd^ 24 h	≤ 1.012	0.74	90.6	48.8	68.5	39.7 (29)	28.8 (21)	27.4 (20)	4.1 (3)	≤ 2
	USG spot afternoon	≤ 1.012	0.92	100.0	64.9	82.2	49.3 (36)	32.9 (24)	17.8 (13)	0 (0)	≤ 2

^*^Accuracy is the percentage of TP and TN samples under the displayed threshold. TP (True Positive), TN (True Negative), FP (False Positive), and FN (False Negative) are provided as numbers; the combined outcome equals *n* = 73 for each line. The accuracy assessment was based on the cut-off of ≤ 1.012 for USG, to determine a low vs high urine concentration. The Uc cut-off represents the urine color in the Printed and 3D Uc models associated with the best fit Uc cut-off to classify urine specific gravity below and above the selected diagnostic standard (≤ 1.012)

In the afternoon, USG Afternoon Print AUC was 0.78, with an accuracy of 75.3%, vs. Afternoon 3D AUC of 0.74 and an accuracy of 68.5%. Finally, for the spot USG samples, the highest accuracy was reported without differences between Printed AUC 0.91 and accuracy 83.6% vs. 3D Uc model, with AUC of 0.92 and accuracy of 82.2%.

## Discussion

Uc scores differed slightly between tools (Printed and 3D Uc model) in the morning but not in the afternoon. Additionally, Uc assessments were slightly more accurate in the afternoon vs. the morning in determining low vs. high urine. No statistical differences for 24-h urine concentration were found between collections, but USG spot morning samples had a significantly higher concentration than the afternoon samples. The Uc charts presented a moderate to strong positive correlation in the morning and the afternoon. Finally, the Uc assessment of spot USG samples yielded the highest accuracy regardless of the Uc scoring method.

In the present study, the method used (Printed or 3D Uc model) did not substantially affect the AUC and the calculated accuracy values for classifying samples for a low vs. high urine concentration against the selected cut-offs, but the time of day did.

The Uc can be of great importance for practitioners and athletes, since it is an easy alternative to assess the hydration status when more accurate methods are not available. In the present study, when comparing both instruments, we found no statistical difference in the afternoon (*p* = 0.07), but a significant difference in the morning (*p* < 0.001). Interestingly, both methods reported the same median Uc score (2). Therefore, the statistical difference was the result of a difference in the distribution of data around the median (based on the 25th and 75th percentiles), indicating that the 3D urine color model more often reported higher scores than the printed Uc chart. These results contrast with those found by Perrier et al. [19] who identified the Uc score 4 as the best cut-off for identifying a urine osmolality > 500 mOsm/kg, but colors may have been appearing darker as a larger volume was scored at less bright light conditions. Also, the current outcome was slightly different from Armstrong et al. [8], who reported that a Uc of 3 or lower was comparable to a mean urine osmolality and USG less than 520 mOsm/kg and 1.014, respectively. Although the AUCs for the 24-h sample comparison, ranging from 0.59–0.78, were lower than previously reported (0.94) [19], the spot morning and afternoon samples yielded higher AUCs than those reported earlier [19], indicating that the methods can accurately determine a low vs. high urine concentration itself. Similarly, a new method to assess Uc in a toilet bowl yielded AUC values of 0.76 using USG, whereas the current study reported AUCs for spot urine samples ranging from 0.81–0.92 [18].

The collection of a 24-h urine sample has been considered a gold standard method to assess hydration status. However, it is cumbersome as it is necessary to collect all voids for a long period of time, especially for athletes, because of their training routine. It has been suggested that the concentration of spot urine samples collected after noon is equivalent to 24-h urine concentration in free-living healthy young adults [10]. In the current study, the afternoon Uc assessment reported a higher AUC than the morning assessment, but for the USG spot sample, the AUC difference was smaller (see Table 3). In this well-hydrated population, while applying lower cut-off values for urine concentration (i.e., ≤ 1.012 USG, which should reflect ~500 mmol.kg^-1^), there does not seem to be a large difference in the accuracy based on Uc assessment to determine the 24-h urine concentration below and above the selected cut-off values. As shown in Table 3, the outcomes of the ROC analyses resulted in some variability for Uc cut-offs, which is indicative of the methods reporting slightly different. Based on the ROC analyses, as well as the significant mean difference between methods in the morning, and the reported bias in the Bland–Altman plot, we believe that the 3D urine color model tends to report differently from the printed Uc chart, specifically when samples have a higher urine concentration, but future research needs to confirm this hypothesis.

The cut-off value selected for this study (i.e., ≤ 1.012 USG) shows that the Uc model assessments effectively help to determine a low urine concentration, suggesting euhydration [19, 22]. The samples collected in the current study were equally distributed around the selected cut-offs, making this data collection extremely suitable for determining the accuracy of these Uc models to identify a low urine concentration. Similar accuracy has been shown in previous studies when applying cut-offs for higher urine concentrations (i.e. ≤ 1.020 USG) [3, 9, 23, 24], similar to the accuracy found in the current study, as shown in Supplementary Table 1. However, it is important to interpret these results cautiously, as the number of samples around the cut-off was limited. 58.9–56.2% of the samples were below 1.012 for USG(1st and 2nd 24 h, respectively), whereas 87.7% of the samples were below 1.020 for USG (for both 1 st and 2nd USG 24 h). Earlier, our research group developed a new 7-color urine chart using higher cut-offs (i.e., ≤ 1.020 USG) and found AUC values of 0.74 and 0.85 when using osmolality and spot samples, respectively [9]. The AUC values in the present study using higher cut-offs were similar to those using lower cut-offs when considering the USG spot: 0.79—0.92, but higher when using osmolality (0.59—0.68). Specifically, regarding the 3D Uc model, the AUC values were similar to those of the Print Uc chart version.

The clinical significance of a one-shade difference in Uc scoring outcome has been best illustrated by a study from our lab describing the within-person reliability of scoring artificial urine colors. The study showed that athletes scored the color or a sample up to 5 shades different, but when ratings were compared with a cut-off value of ≥ 4, indicating under- or hypohydration, most of the samples that were tested, despite their test–retest variation, would still be classified as similar or darker than this cut-off color, resulting in theoretically correct classification of the sample to reflect a “dark urine” [14]. In fact, as soon as scores hover around the selected urine color cut-off value, it is more likely that misclassifications occur, which implicates the need to standardize and optimize urine color scoring by considering container volume as well as light type and intensity [13]. It is important to highlight that, in the present study, the samples were analyzed within one hour after collection, while sitting in a thermoneutral environment (20–25 °C). The Uc was evaluated by the participants within ~ 20 min, since previous literature showed that Uc is affected by storage conditions and time [16].

### Strengths and limitations

The current study held some clear strengths. Most of the data were collected on wildland firefighters, a profession that benefits from having a good hydration status, including a fair number of females (12%), which exceeded the common average female representation of 9% of females in the fire service [16]. Collecting 32 h of urine allowed us to directly relate the Uc assessment in the morning and in the afternoon to the 24 h of urine collection leading up to that specific assessment.

The study also included some limitations. Participants presented a better hydration status than initially expected in this cohort. They might have changed their behavior regarding fluid intake patterns because they were being evaluated on hydration status. This resulted in a distribution of the data around the lower cut-off value instead of the higher cut-off value (and the number of samples with a concentration below the lower cut-off and above the higher cut-off) as this may influence the relevance (and generalization) of the cut-off values proposed (i.e., the lower cut-off is providing a much better two-sided distribution around the cut-off with around 50% below and above the cut-off in comparison to the higher cut-off value, making the outcome for the higher cut-off value potentially less reliable.

During the pilot tests, it was noticed that the 3D color solution created some sediments over time. However, a gentle shake reconstituted the solution to its original status. Carrying a 3D model may be an additional logistical layer to urine color assessment. However, in its use it will not be different from a traditional color chart. The current 3D model, whose recipe was included in the manuscript, could be created in a simple way, and hopefully this will inspire others to develop more user-friendly 3D models.

Future studies could evaluate the validity and accuracy of the new 3D Uc model in different conditions, such as a) a wider spectrum of urine concentration, as the current study could only reasonably assess the accuracy of this method in urine samples with a relatively low concentration because of the data collected, b) different light conditions, and c) verify the actual volume of pigment needed to create the 3D color model vs. drops of pigments performed in this study.

### Practical applications

It is known that the determination of Uc depends on several factors, such as the material of the chart, type of container, volume, and light conditions [9, 13, 14, 25]. In the present study, no clear difference was seen between the printed and newly developed 3D Uc model, but specifically when users cannot control for light conditions or when light conditions for consecutive Uc assessments may differ, the 3D Uc model may provide a good alternative for the printed Uc chart. Overall, the 3D Uc model does not substantially outperform the Printed version in evaluating urine concentration.

## Conclusions

The new 3D Uc model is a valid and accurate tool to identify low vs high urine concentration comparable to a 7-color Uc chart. The Printed chart and 3D Uc model presented similar scores in the morning and afternoon but with slightly higher accuracy in classifying low vs. high urine concentration in the afternoon.

## Supplementary Information

Supplementary Material 1.

## Data Availability

The dataset is available upon reasonable request.

## Abbreviations

ASU: Arizona State University
AUC: Area Under the Curve
DHS: Department of Homeland Security
FN: False Negative
FP: False Positive
IRB: Institutional Review Board
IQR: Interquartile Range
LED: Light Emitting Diode
Max: Maximum
Min: Minimum
N/A: Not Applicable
Osm: Osmolality
RGB: Red, Green and Blue
ROC: Receiver Operator Curves
Uc: Urine Concentration
USG: Urine Specific Gravity
TN: True Negative
True: Positive
3D: Three Dimensional
