# Supplementary material for: A 3D color model reports urine color similarly to a printed color chart with similar accuracy to determine a low vs. high urine concentration
Source: Perform Nutr. 2025 Sep 12;1(1):8. doi: 10.1186/s44410-025-00009-3 (PMC12447702; doi:10.1186/s44410-025-00009-3)
Supplement: Supplementary file 1 — Supplementary Material 1. [file 44410_2025_9_MOESM1_ESM.pdf]

SUPPLEMENTARY MATERIAL

Supplementary Table 1. Receiver operating characteristic evaluation of the Printed and 3D urine color charts and percentage accuracy of correct classification of low vs. high urine concentration using a higher cut-off.

Category	Diagnostic standard	Threshold	AUC	Sensitivity %	Specificity %	Accuracy %*	TP % and n	TN % and n	FP % and n	FN % and n	Uc Cut-off
Morning print	USG 1st 24h	≤1.020	0.75	77.8	39.1	63.0%	9.6(7)	53.4(39)	34.2(25)	2.7(2)	≤3
	USG spot morning	≤1.020	0.82	75.0	50.6	75.3%	28.7(21)	46.5(34)	15(11)	9.5(7)	≤3
Morning 3D	USG 1st 24h	≤1.020	0.68	77.8	40.6	61.6%	9.6(7)	52.1(38)	35.6(26)	2.7(2)	≤3
	USG spot morning	≤1.020	0.79	82.1	59.9	79.5%	31.5(23)	47.9(35)	13.6(10)	6.8(5)	≤3
Afternoon print	USG 2nd 24h	≤1.020	0.82	88.9	23.4	78.0%	11.0(8)	67.1(49)	20.5(15)	1.4(1)	≤3
	USG spot afternoon	≤1.020	0.91	85.7	76.1	89.0%	24.6(18)	64.3(47)	6.8(5)	4.1(3)	≤3
Afternoon 3D	USG 2nd 24h	≤1.020	0.75	66.7	26.6	72.6%	8.2(6)	64.4(47)	23.3(17)	4.1(3)	≤3
	USG spot afternoon	≤1.020	0.93	85.7	76.1	89.0%	24.6(18)	64.3(47)	6.8(5)	4.1(3)	≤3

*Accuracy is the percentage of TP and TN samples under the displayed threshold. TP (True Positive), TN (True Negative), FP (False Positive), and FN (False Negative) are provided as numbers; the combined outcome equals n=73 for each line. The accuracy assessment was based on the cut-off of ≤ 1.020 for USG, to determine a low vs high urine concentration. The Uc cut-off represents the urine color in the Printed and 3D Uc models associated with the best fit Uc cut-off to classify urine specific gravity below and above the selected diagnostic standard (≤ 1.020).
